# Case Report: Gemcitabine-induced membranoproliferative glomerulonephritis with immune complexes in a patient with metastatic pancreatic cancer

**DOI:** 10.3389/fmed.2025.1666859

**Published:** 2025-11-17

**Authors:** Ahmed Abdelhakeem, Lyle Baker, Md. Shahrier Amin, Hani Babiker, Nabeel Aslam, Umair Majeed

**Affiliations:** 1 Department of Medicine, Division of Hematology and Oncology, Mayo Clinic, Jacksonville, FL, United States; 2 Department of Medicine, Division of Nephrology and Hypertension, Mayo Clinic, Jacksonville, FL, United States; 3 Department of Laboratory Medicine and Pathology, Mayo Clinic, Jacksonville, FL, United States

**Keywords:** gemcitabine, membranoproliferative glomerulonephritis, pancreas, thrombotic microangiopathy, proteinuria

## Abstract

Gemcitabine is a widely used chemotherapeutic agent for pancreatic adenocarcinoma, that is associated with rare but serious renal complications including thrombotic microangiopathy (TMA). We report a unique case of biopsy-proven membranoproliferative glomerulonephritis (MPGN) with immune complex deposition in a woman receiving gemcitabine for metastatic pancreatic cancer. She developed new-onset hypertension, proteinuria, microscopic hematuria, and progressive renal dysfunction shortly following initiation of gemcitabine. Extensive autoimmune, complement, paraprotein, and viral serologies were unremarkable aside from a low haptoglobin. Kidney biopsy revealed an MPGN pattern with immune deposits. Gemcitabine was discontinued, and the patient was treated with corticosteroids and kidney-protective therapies targeting blood pressure and proteinuria reduction. Her proteinuria decreased significantly, and her renal function returned back to baseline. This case highlights a rare manifestation of gemcitabine-induced nephrotoxicity with immune complex MPGN, suggesting a possible novel mechanism of drug-associated glomerular injury.

## Background

Gemcitabine is a widely used chemotherapeutic agent for the treatment of various solid tumors, particularly pancreatic adenocarcinoma. Although generally well tolerated, it is associated with several adverse effects, including hematologic toxicities, pulmonary complications, and renal dysfunction. Renal injury related to gemcitabine is thought to be dose-dependent and is primarily mediated by endothelial injury, leading to thrombotic microangiopathy (TMA), with an estimated incidence ranging from 0.015 to 1.4% (1, 2).

Membranoproliferative glomerulonephritis (MPGN) is a histologic pattern of glomerular injury characterized by mesangial and endothelial proliferation and duplication of the glomerular basement membrane. MPGN may arise from several different mechanisms, including immune complex deposition, complement dysregulation, or endothelial injury without detectable immune deposits. It is typically associated with chronic infections, autoimmune disorders, and monoclonal gammopathies.

To date, only one documented case has reported MPGN with electron-dense deposits in a patient receiving gemcitabine, though causality was unclear due to concurrent vinorelbine therapy for peritoneal mesothelioma (3). To our knowledge, no prior cases have described immune complex-mediated MPGN directly attributed to gemcitabine monotherapy.

We present a rare case of biopsy-proven immune complex MPGN with concurrent TMA features in an elderly woman receiving gemcitabine maintenance monotherapy for metastatic pancreatic adenocarcinoma, highlighting the importance of recognizing atypical patterns of drug-induced nephrotoxicity and the role of early kidney biopsy in guiding management.

## Case presentation

An elderly female in her 70s with a history of osteoporosis presented to our institution with a new diagnosis of pancreatic adenocarcinoma, with metastatic involvement of the liver, bone, and peritoneum. Initial laboratory evaluation revealed elevated carbohydrate antigen 19–9 (CA 19–9) at 1151 U/mL, baseline serum creatinine of 1.0–1.1 mg/dL, an estimated glomerular filtration rate (eGFR) of 52–59 mL/min/BSA, and normal liver function tests. Genetic testing with Boston Gene platform for pancreatic cancers showed KRAS G12D and EGFR alterations.

The patient was offered standard-of-care chemotherapy with FOLFIRINOX or enrolment in a clinical trial consisting of protein-bound paclitaxel, cisplatin, and gemcitabine in combination with tumor treatment fields. She elected to enroll in the clinical trial. After three cycles of combination chemotherapy per clinical trial protocol, she developed acute kidney injury (AKI) stage 1, with serum creatinine rising to 1.42 mg/dL. Cisplatin was suspected of being the offending agent, and its dose was reduced from 30 mg/m^2^ to 20 mg/m^2^. Following dose adjustment, her kidney function improved, and creatinine returned to baseline (1.0–1.1 mg/dL). She subsequently completed six total cycles of trial-based chemotherapy.

She then transitioned to gemcitabine maintenance therapy, during which her kidney function remained stable for approximately 5 months. At month six, her serum creatinine began to rise, without improvement despite intravenous fluid administration with each gemcitabine cycle. By month seven, she developed AKI stage 2, with a peak creatinine of 2.09 mg/dL. She was referred to the nephrology clinic for further evaluation.

## Investigations

At the time of nephrology evaluation, the patient reported foamy urine and was noted to have new-onset systolic hypertension ranging 140–160 mmHg, compared to her prior baseline of 110–120 mmHg. Urinalysis demonstrated microscopic hematuria, with 32 red blood cells per high-power field. Quantitative testing for proteinuria revealed a random urine protein-to-creatinine ratio ranging from 1.46 to 2.94 mg/mg, and an albumin-to-creatinine ratio of 685 mg/g, consistent with subnephrotic-range proteinuria.

To evaluate for underlying autoimmune, infectious, or paraprotein-related kidney disease, a comprehensive non-invasive serologic workup was performed. This revealed a low haptoglobin level of 25 mg/dL (normal 30–200 mg/dL), suggestive of possible microangiopathic hemolysis. Peripheral blood smear was without schistocytes. All other serologic studies were within normal limits, including serum protein electrophoresis, serum free light chains, cryoglobulins, antinuclear antibody, anti-double-stranded DNA, extractable nuclear antigens, myeloperoxidase and proteinase 3 antibodies, complement levels (C3 and C4), creatine kinase, and HIV, hepatitis B and C serologies. Renal ultrasound showed no hydronephrosis or evidence of obstruction.

**Figure 1:**
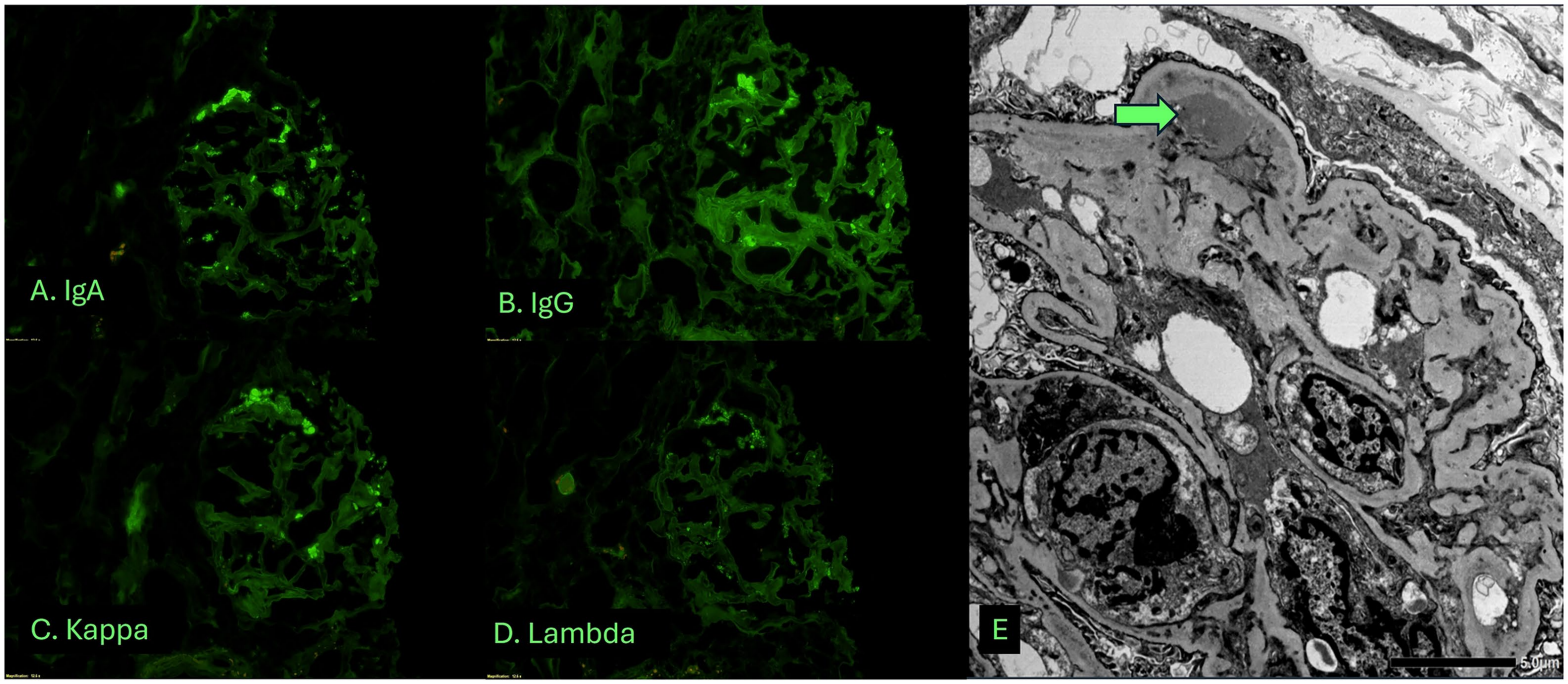
Light microscopic examination of the biopsy shows a membranoproliferative pattern glomerulonephritis associated with tubulointerstitial fibrosis and atrophy. **(A)** Low magnification view of biopsy cores stained with Hematoxylin and eosin stain. **(B–E)** High magnification view of glomeruli showing membranoproliferative pattern glomerulonephritis. **(B)** H&E stain showing two glomeruli with accentuated lobularization. Note RBC casts in tubules. **(C)** Jones methenamine silver stain. **(D)** Periodic acid Schiff stain. **(E)** Masson’s trichrome stain.

**Figure 2:**
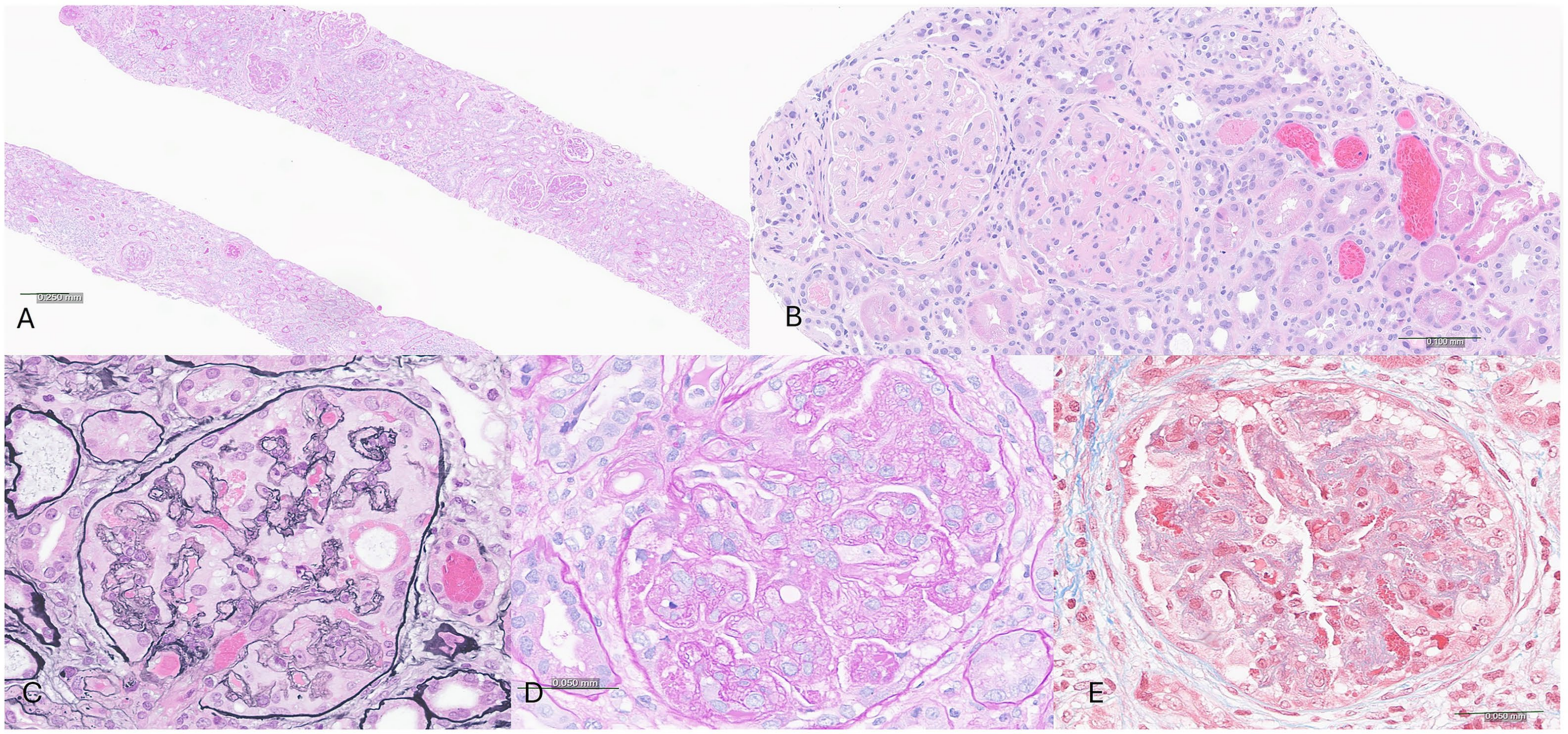
Immunofluorescence and electron microscopic studies showing polyclonal, capillary loop and mesangial deposits. **(A–D)** Immunofluorescence studies highlighting a glomerulus stained with IgA **(A)**, IgG **(B)**, kappa light chain **(C)** and lambda light chain **(D)**. **(E)** Electron microscopy confirms the membranoproliferative pattern with double contours, hypercellularity, and shows subendothelial deposits (arrow).

Given her progressive decline in kidney function, new-onset hematuria and proteinuria, and the absence of a systemic cause, a kidney biopsy was obtained. Adequate renal cortical parenchyma was present with 50 glomeruli present, 14 of which were globally scleortic. The glomeruli showed a predominantly membranoproliferative pattern of injury with accentuated lobularization, mesangial hypercellularity and extensive glomerular basement membrane duplication. Occasional pseudo thrombi were noted within some capillary loops. Focal (up to 5) glomeruli showed cellular or fibrocellular crescents and some of the sclerotic glomeruli showed evidence of crescents. The interstitium showed a patchy lymphoplasmacytic infiltrate limited to areas with interstitial fibrosis and tubular atrophy in approximately 30–40% of the parenchyma. The blood vessels were unremarkable and no vasculitis was noted (Figure 1). Immunofluorescence demonstrated 1–2 + granular staining for IgA, IgM, IgG, C3, C1q, kappa, and lambda light chains in segmental capillary loops and mesangial areas (Figure 2). There was no significant tubulointerstitial staining. Kappa and lambda stained equally in the deposits and tubular casts. Electron microscopy confirmed the extensive membranoproliferative pattern of injury with extensive double contour formation in most capillary segments, associated with mesangial cell interposition and prominent endothelial cells. Occasional subendothelial and few mesangial electron-dense deposits could be appreciated, none showing any particular ultrastructure. No deposits were seen in the tubulointerstitial areas (Figure 2).

## Differential diagnosis

The kidney biopsy findings supported drug-induced thrombotic microangiopathy (TMA), but the presence of some immune deposits by immunofluorescence and electron micrsocopy raised the possibility to consider differential diagnoses including cryoglobulinemic glomerulonephritis, autoimmune disorders such as lupus nephritis, monoclonal gammopathy-related kidney disease, hepatitis-associated MPGN, malignancy, and idiopathic immune complex-mediated MPGN.

MPGN is a histopathologic pattern of injury that may result from immune complex deposition, complement dysregulation, or non-immune endothelial injury, such as chronic TMA. In this case, the presence of immune complex deposition and electron-dense deposits supported an immune complex-mediated process.

Cryoglobulinemic glomerulonephritis was considered due to its association with immune complex MPGN and the potential for thrombus-like capillary deposits. However, this was considered unlikely given the absence of systemic features of cryoglobulinemia, a negative cryoglobulin screen, and amorphous nature of the immune deposits without any sub-structure. Although “full house” staining is commonly seen in lupus nephritis, the patient had no clinical features of systemic lupus erythematosus, and her autoimmune serologies were unremarkable. These findings made lupus and autoimmune glomerulonephritis unlikely. Hepatitis B and C are well-known triggers of immune complex MPGN, but both were excluded with negative viral hepatitis panels. Monoclonal gammopathy–associated MPGN was also considered, but serum protein electrophoresis with immunofixation and free light chains ratio were normal, and the immunofluorescence pattern of equal intensity kappa and lambda staining was not supportive of monoclonal gammopathy induced MPGN.

**Figure 3:**
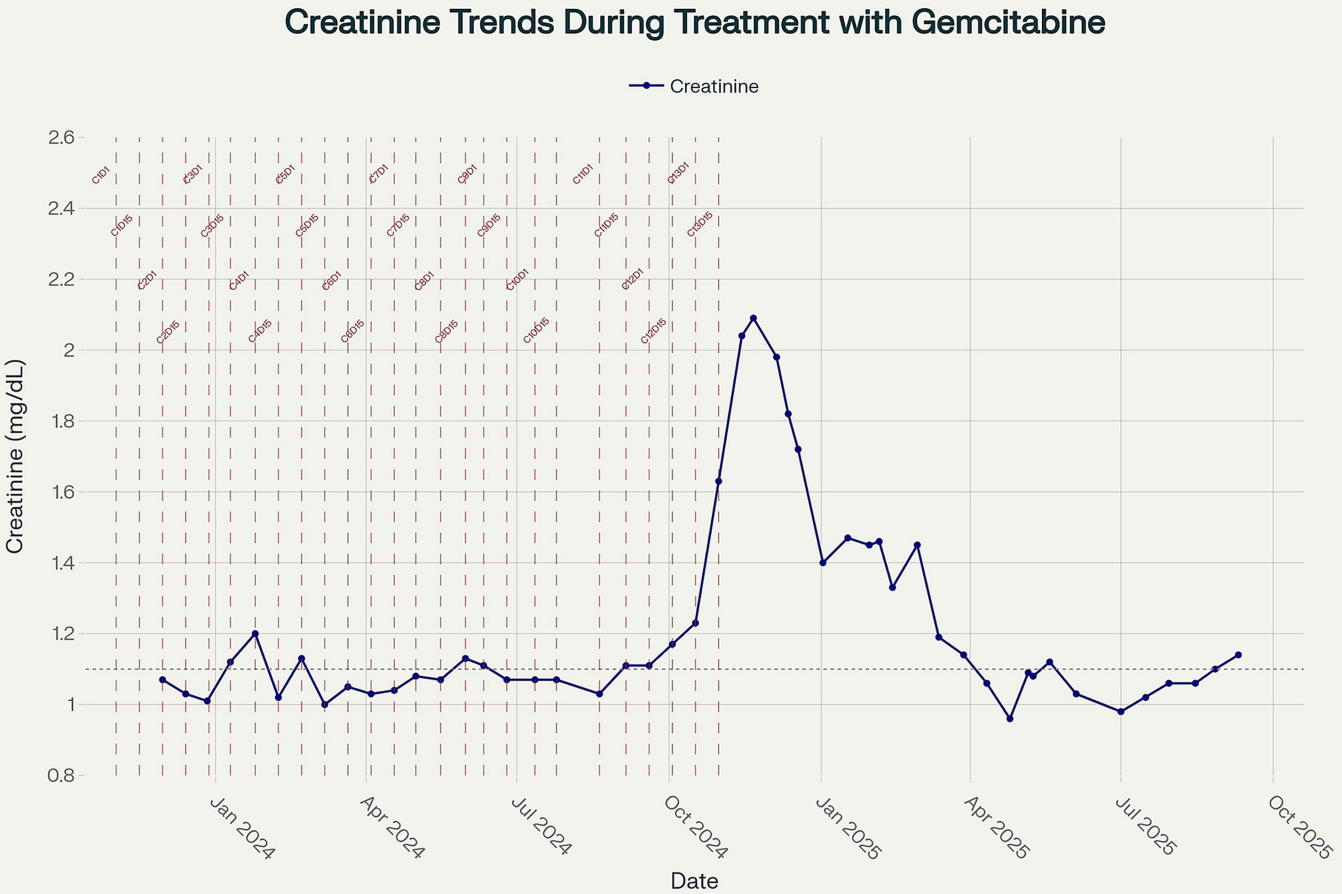
Line plot showing longitudinal serum creatinine measurements (mg/dL) from November 2023 to September 2025. Vertical dashed lines indicate the timing of gemcitabine administration cycles, with cycle numbers annotated. The dotted horizontal line represents the upper limit of normal for creatinine (1.04 mg/dL). Creatinine values remained within or near the normal range during the early treatment period and subsequently showed a progressive rise after cycle 13, peaking in November 2024, before partially improving upon treatment discontinuation.

In the absence of an identifiable autoimmune, infectious, or paraprotein-related cause – and given the temporal relationship with gemcitabine monotherapy (Figure 3), presence of pseudo thrombi, new-onset hypertension, proteinuria, and hematuria – the most likely diagnosis was gemcitabine-induced immune complex MPGN. Immune dysregulation can be triggered by malignancy with resultant immune complex glomerulonephritis, although pancreatic cancer has been reported to be associated with membranous nephropathy (4), there are no reported cases of pancreatic cancer induced MPGN. In addition, the improvement of MPGN manifestations in our case with withdrawal of gemcitabine argues against the paraneoplastic immune complex mediated MPGN.

## Treatment

In light of the biopsy findings and the suspected diagnosis of gemcitabine-induced immune complex MPGN, the patient was withdrawn from the clinical trial, and gemcitabine therapy was permanently discontinued.

She was initiated on oral corticosteroid therapy with prednisone 40 mg daily (approximately 1 mg/kg) for 7 days, followed by a tapering regimen over the subsequent 4 weeks before discontinuation. This regimen was chosen to target the suspected immune-mediated component of her glomerular process. Additionally, she was started on valsartan, which was gradually titrated upward to optimize both blood pressure control and proteinuria reduction. In the weeks following discontinuation of gemcitabine and initiation of corticosteroid therapy, the patient’s renal function improved, with serum creatinine returning to her baseline of 1.0–1.1 mg/dL.

## Discussion

Gemcitabine is a nucleoside analogue chemotherapy agent widely used in the treatment of various solid tumors, including pancreatic adenocarcinoma. While its most common adverse effects are hematologic, pulmonary, and gastrointestinal, renal toxicities - though relatively rare – have been increasingly recognized. Reported renal complications include proteinuria, hematuria, acute kidney injury, and hemolytic uremic syndrome (HUS), the latter representing a manifestation of thrombotic microangiopathy (TMA) (5).

The pathogenesis of gemcitabine-induced renal injury is thought to be dose-dependent and primarily mediated by endothelial injury, leading to TMA (5, 6). In most reported cases, kidney biopsies show classic features of TMA without immune complex deposition. Clinical manifestations are often delayed, emerging after multiple treatment cycles, and may include new-onset hypertension, proteinuria and hematuria, and a progressive decline in renal function (3, 7, 8).

Our case contributes to the limited literature on gemcitabine-associated glomerular injury by describing a biopsy-confirmed case TMA with some immune deposits. MPGN is a histologic pattern that can result from several pathogenic mechanisms, including immune complex deposition, complement dysregulation or endothelial injury without immune deposits. While gemcitabine is a recognized cause of renal-limited TMA, to our knowledge, this is the first reported case of MPGN with immune complex deposition temporally and mechanistically linked to gemcitabine monotherapy.

We believe this case represents a rare histopathologic variant where in addition to the endothelial injury, there are also immune complexes that aggravate the glomerular disease as evidenced by presence of immune deposits by both immunofluorescence and electron microscopy and occasional crescents in this patient. We hypothesize that in our patient, gemcitabine elicited an immune response with generation of autoantibodies analogous to drug induced lupus nephritis (9) which is supported by the presence of “full house pattern” on immunofluorescence microscopy in our case. Absence of detectable autoantibodies in blood does not rule out presence of drug induced autoimmunity as there have been reported cases of ANA negative patients with kidney biopsy showing full house pattern lupus nephritis (10) and there may be a rare yet to be discovered gemcitabine induced autoantibody triggering this response. Alternatively, one may also hypothesize that gemcitabine-induced endothelial injury may have either triggered secondary immune complex deposition in the glomeruli or unmasked an underlying latent glomerular process. Whether the immune complexes are a direct consequence of drug-induced antigenicity, a parainflammatory epiphenomenon, or an unrelated coincident pathology remains uncertain.

From a clinical standpoint, this case underscores the importance of routine renal monitoring in patients receiving gemcitabine and the need for early nephrology evaluation in the presence of new-onset hypertension, haematuria, or proteinuria. In select cases, renal biopsy is essential for establishing a definitive diagnosis and guiding treatment decisions. Discontinuation of gemcitabine and initiation of supportive therapy – including renin-angiotensin system blockade and corticosteroids – led to recovery of renal function and improvement in proteinuria.

In conclusion, this case expands the spectrum of gemcitabine-associated nephrotoxicity and highlights a potentially novel glomerular injury pattern.

## Data Availability

The raw data supporting the conclusions of this article will be made available by the authors, without undue reservation.
